# Virome assembly and annotation in brain tissue based on next‐generation sequencing

**DOI:** 10.1002/cam4.3325

**Published:** 2020-08-01

**Authors:** Zihao Yuan, Xiaohua Ye, Lisha Zhu, Ningyan Zhang, Zhiqiang An, W. Jim Zheng

**Affiliations:** ^1^ School of Biomedical Informatics University of Texas Health Science Center at Houston Houston TX USA; ^2^ Texas Therapeutics Institute Institute of Molecular Medicine McGovern Medical School University of Texas Health Science Center at Houston Houston TX USA

**Keywords:** assembly, GBM, metagenomics., virosphere

## Abstract

The glioblastoma multiforme (GBM) is one of the deadliest tumors. It has been speculated that virus plays a role in GBM but the evidences are controversy. Published researches are mainly limited to studies on the presence of human cytomegalovirus (HCMV) in GBM. No comprehensive assessment of the brain virome, the collection of viral material in the brain, based on recently sequenced data has been performed. Here, we characterized the virome from 111 GBM samples and 57 normal brain samples from eight projects in the SRA database by a tested and comprehensive assembly approach. The annotation of the assembled contigs showed that most viral sequences in the brain belong to the viral family Retroviridae. In some GBM samples, we also detected full genome sequence of a novel picornavirus recently discovered in invertebrates. Unlike previous reports, our study did not detect herpes virus such as HCMV in GBM from the data we used. However, some contigs that cannot be annotated with any known genes exhibited antibody epitopes in their sequences. These findings provide several avenues for potential cancer therapy: the newly discovered picornavirus could be a starting point to engineer novel oncolytic virus; and the exhibited antibody epitopes could be a source to explore potential drug targets for immune cancer therapy. By characterizing the virosphere in GBM and normal brain at a global level, the results from this study strengthen the link between GBM and viral infection which warrants the further investigation.

## Graphical abstract

We employed a data driven approach to identify virus species from the GBM NGS sequencing data. These virus could be potential novel therapeutic targets for cancer therapy.

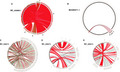

## 1 BACKGROUND

In 2019, an estimated 86 970 new cases of brain and other central nervous system (CNS) tumors are expected to be diagnosed in the United States alone.^1^ It was projected that 47.7% of primary malignant brain tumors are glioblastoma multiforme (GBM)—one of the most killing tumors with a 5‐year survival rate less than 6% and a 12‐15 months median survival time even with the most advanced treatment.^2^, ^3^, ^4^, ^5^, ^6^ Although there is rapid advancement in cancer research and therapies, outcomes for GBM patients remain dismal due to the lack of knowledge of GBM etiology. GBM is not usually inherited^7^ and the causes of GBM have always been a topic of controversy. Hypothesized causes of GBM include exposure to ionizing radiation,^8^ use of electronics,^8^, ^9^, ^10^, ^11^ or viral infections.^12^, ^13^, ^14^

Viruses have been identified as important factors in the incidence of various cancers.^15^, ^16^ Many efforts have been devoted to detect the cancer causing virus or design oncolytic virus for tumor treatment.^17^, ^18^ For example, a novel Merkel cell polyomarvirus was discovered in Merkel cell carcinoma,^19^, ^20^ and the herpes virus Epstein‐Barr virus (EBV) was identified from the large B‐cell lymphomas,^21^ Burkitt's lymphomas,^22^ and gastric carcinoma.^23^ In addition, the human papillomaviruses (HPV) have been proven to play essential roles in promoting oncogenesis in cervical carcinoma.^16^ The Hepatitis B virus (HBV) and its integrations were also identified as a major risk factors for the development of hepatocellular carcinoma.^24^, ^25^, ^26^, ^27^ Furthermore, there have been studies focusing on identifying insertion sites of viruses in the human genome from next‐generation sequencing data in the Cancer Genome Atlas (TCGA).^16^, ^28^ These studies clearly demonstrate the importance of investigating the association between viruses and cancer development.

Since 2002, there have been significant efforts to investigate the correlation between human cytomegalovirus (HCMV)^12^ and GBM occurrence by different methods such as polymerase chain reaction, in situ hybridization, immunohistochemistry, and next‐generation sequencing. Despite of these efforts, the presence of HCMV as well as other herpes virus in brain and their correlation with the development of GBM remains an area of controversy.^12^, ^14^, ^16^, ^29^, ^30^, ^31^, ^32^, ^33^, ^34^, ^35^, ^36^, ^37^, ^38^, ^39^, ^40^, ^41^, ^42^, ^43^, ^44^, ^45^, ^46^, ^47^, ^48^, ^49^, ^50^, ^51^, ^52^, ^53^

In addition to HCMV, some studies observed the presence of human papillomavirus (HPV) and hepatitis B in low‐grade gliomas (LGG)^52^ from next‐generation sequencing data. In these studies, short sequence reads were aligned to the reference viral genome sequences to identify these viruses. One limitation of such approach is the high false positive results due to the congregation of short reads in highly repetitive regions, or in the regions that contain artificial sequences in some of the reference genomes.^54^ In addition, traditional approaches had only identified 4021 characterized virus species according to Baltimore virus classification,^55^ which only represent a tiny fraction of the virome diversity. Furthermore, a large number of unknown reads that cannot be mapped to any reference genome are discarded. Therefore, current approach does not provide a full depiction of the landscape of the virome in the brain, and a comprehensive assessment of the virome and its correlation to GBM is needed.

The assembly of the metagenomics is vitally important to the quality of viral detection. However, assembly of the viral genome has always been challenging due to the fast evolving and fragmented nature of the viral genome.^56^, ^57^ In recent decades, several metagenomic assemblers have been designed for the assembly of different sequencing data.^58^, ^59^, ^60^ The assembly software with long k‐mer length can generate contigs more accurately by reducing chimeric sequences.^61^ In addition, the annotation of the assembly directly against a reference sequence database via BLAST is an easy and effective approach to characterize sequences.^62^

In this study, we applied metagenomics approach to characterize the virosphere of GBM at a global scale and observed some novel viruses previously isolated only from nonhuman organisms. We also observed that the contigs matching the genome sequences of the herpes virus only make up a small portion of the whole viral genome. In addition, we identified some novel sequences with no known annotations. Further analysis showed that these sequences have the signature for antibody epitopes. These findings will provide novel avenues toward future GBM research and therapies.

## 2 METHODS

### 2.1 Data source and availability

We searched the NCBI Sequence Read Archive (SRA: https://www.ncbi.nlm.nih.gov/sra), Gene Expression Omnibus (GEO: https://www.ncbi.nlm.nih.gov/geo/), and PubMed literature to collect NGS studies relating to GBM and normal brain tissues. We also identified a set of samples infected with known viruses as our “positive controls” to test if our assembly approaches can detect these viruses from the sequencing data. We limited our study to the data generated from Illumina sequencing platform and the RNA‐seq data were downloaded from SRA database. The list of accessions for the source data are shown in Supplemental File 1.

### 2.2 Positive controls and brain sample assembly

The raw reads in each study were first trimmed and checked using Trimmomatic (version 0.36)^63^ and fastqc.^64^ Ambiguous nucleotides (N’s), extreme short reads (<30 nt), and low‐quality bases were trimmed with a sliding window size of 4. The reads were then mapped to the human genome (GRCh38.p13) via STAR.^65^ Reads that cannot be mapped to human genome were collected for further analysis.

**FIGURE 1:**
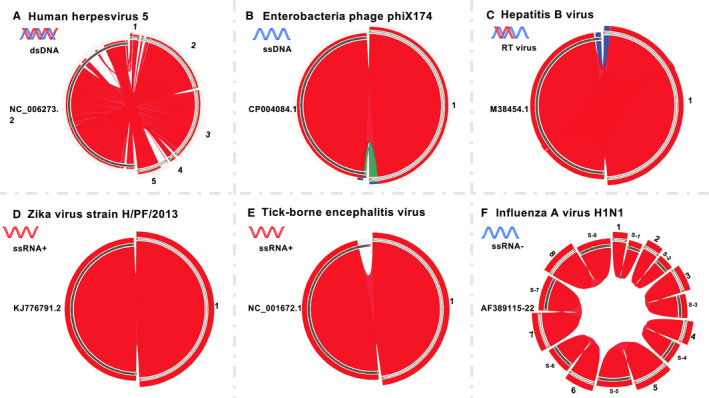
The assembled contigs from known viral infections and synteny analysis with their reference genomes. A, Human herpesvirus 5, reference genome accession: NC_006273 contigs: 1. k89_1468; 2. k89_1723; 3. k89_1974; 4. k89_821; 5. k89_887. B, Enterobacteria phage phiX174 reference genome accession: CP004084.1 contigs: 1. k141.3724. C, Hepatitis B virus reference genome accession: M38454.1 contigs: 1. k141.13661. D, Zika virus strain H/PF/2013 reference genome accession: KJ776791.2 contigs: k95.45717. E, Tick‐borne encephalitis virus reference genome accession: NC.001672.1 contigs: k79.90. F, Influenza A virus (A/Puerto Rico/8/34/Mount Sinai(H1N1)) reference genome accession: S‐1: ENA.AF389122.AF38912; S‐2: ENA.AF389121.AF38912; S‐3: ENA.AF389119.AF38911; S‐4: ENA.AF389120.AF38912; S‐5: ENA.AF389115.AF38911; S‐6: ENA.AF389118.AF38911; S‐7: ENA.AF389116.AF38911; S‐8: ENA.AF389117.AF38911; contigs: 1. k59.54; 2. k59.36; 3. k59.42; 4. k59.46; 5. k59.58; 6. k59.53; 7. k59.41; 8. k59.56

**FIGURE 2:**
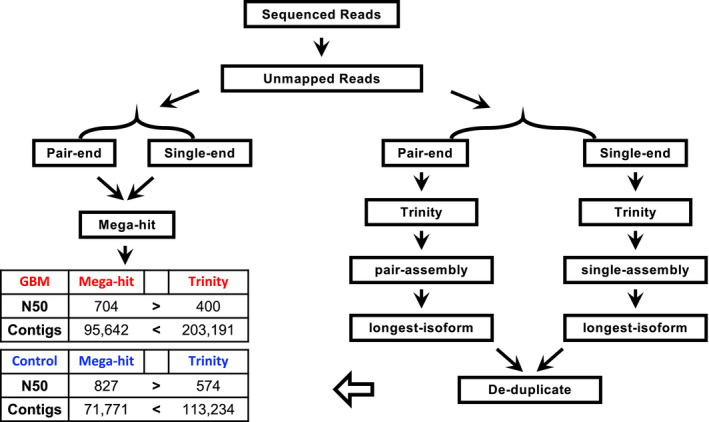
The assembly approach used for GBM and normal brain RNA‐seq dataset

For the samples with known virus infections, the MEGAHIT was used for contig assembly, and the resulting contigs were compared with the reference viral genomes (Figure 1). For brain RNA‐seq data, viral sequences were detected by the pathogen discovery program, READSCAN.^66^ A read is considered as a viral sequence if it covers at least 10% of the reference genome of the virus. The assembly of the viral sequences was conducted with MEGAHIT and Trinity, and their assembly results are compared and evaluated (Figure 2). The pair‐end and single‐end reads were pooled and assembled by MEGAHIT.^67^, ^68^ Trinity is also an efficient and robust software for de novo assembly of transcriptomes from RNA‐seq data, and was also used for the assembly. The pair‐end and single‐end reads were assembled separately. The longest isoform for each gene assembled was selected using get_longest_isoform_seq_per_trinity_gene.pl. In order to reduce redundancy, the assembly was then processed by CD‐Hit (version 4.5.4) to remove duplicated contigs.^69^ The threshold of sequence identity was set at 1.0, with the alignment coverage greater than 90% of the shorter sequence, and word length of 5.

### 2.3 Viral contig annotation with RefSeq database

The contigs with length over 500 bp were annotated to known viruses references in both protein and nucleotide databases at NCBI via BLAST^70^ and Diamond^71^ with the cutoff of e‐value < 1e‐10. For “positive controls,” the annotated virus contigs and its synteny with the virus genome were visualized with Circos using tBLASTN. Ribbons are colored based on the E‐value, with red represents the best hit.^72^

The number of reads contributed to the assembly of each “viral” contig from each sample was calculated to ensure the assembly quality (Figure 4) by mapping to the “viral” contigs using Bowtie2^73^ and viewed by Tabular.^74^ The charts were generated using the R ggplot package.^75^

### 2.4 Novel contigs annotation and characterization

**FIGURE 3:**
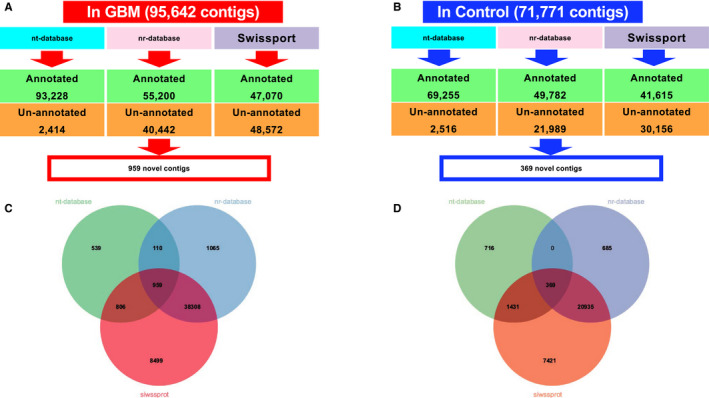
The annotation results from nt‐database, nr‐database, and Swiss‐Prot databases. The annotation results for A, GBM and B, normal brain. The overlap of the unknown annotations in C, GBM and D, normal brain

The shared contigs that have no annotation from the above analysis are view in Venn diagram (Figure 3).^76^ The unknown contigs are extracted and the phylogenetic tree was built using Fast tree (version 1.0.1).^77^ The potential viral open reading frames (ORFs) were predicted by ORF finder (https://www.ncbi.nlm.nih.gov/orffinder/). The minimal ORF length was set as 75, with any sense codon and standard genetic code applied. For each of the putative protein‐coding contigs, we applied TMHMM Server v. 2.0 to predict transmembrane domains.^78^ Antibody epitope prediction was conducted by Bepipred Linear Epitope Prediction method in Immune Epitope Database (IEDB) (https://www.iedb.org/home_v3.php).^79^, ^80^, ^81^, ^82^ In order to ensure the quality of each contig, we calculated the reads coverage for each sample in the samtools,^83^ and only kept those contigs with the coverage over 60% of its entire length for analysis.

## 3 RESULTS

### 3.1 Assembly of positive controls

To validate our approach, we tested six samples with known viral infections as positive controls to evaluate our methods for viral sequence assembly. These six samples include Human herpesvirus 5 (double stranded DNA virus), Enterobacteria phage phiX174 (single stranded DNA virus), HBV (double stranded DNA virus with reverse transcription), Zika virus (single‐stranded, positive‐sense RNA virus), Tick‐borne Encephalitis virus (single‐stranded, positive‐sense RNA virus), and Influenza A virus H1N1 (fragmented, single‐stranded, negative‐sense RNA virus). These viruses cover major categories of different types of viruses to ensure the validity of our approach.

After trimming and mapping the reads to human genome, the unmapped high‐quality reads from the positive controls were assembled via MEGAHIT. The assembly results from each virus infected samples were compared to its corresponding reference sequences. We observed that for each positive control, the assembled contigs can cover over 90% of the reference genome of the corresponding virus (Figure 1). Five assembled contigs from the human herpesvirus 5 virus sample cover more than 90% of the viral genome (Figure 1A). One assembled contig from phage X174, HBV, zika, and Encephalitis samples each covers more than 90% of the corresponding viral genome (Figure 1B‐E). Furthermore, eight contigs from Influenza A virus infected sample can cover the eight segments of the influenza A H1N1 reference genome, respectively (Figure 1F). These results showed that our assembly approach is suitable and reliable for the metagenomic studies.

### 3.2 Brain RNA‐Seq reads assembly

We collected 111 GBM and 57 healthy brain data sets from eight different projects. This large number of datasets ensures the quality of contig assembly (Supplemental 2). In total, there are 6609 M (Million) raw sequencing reads for GBM and 2681 M for healthy brain. The low‐quality reads and reads that map to human genome were then removed to yield 210.0 M high quality reads for GBM and 115.4 M for health brain. For each group, reads were pooled together and assembled with MEGAHIT and Trinity, respectively. Using N50, N90 and the number of contigs as a criteria, MEGAHIT performed better than Trinity in assembling the GBM RNA‐Seq reads (Figure 2): MEGAHIT generated 95 642 contigs with N50 = 704 bp while Trinity generated 203 191 contigs with N50 = 400bp. In healthy brain, MEGAHIT generated 71 771 contigs with N50 = 827bp while Trinity generated 113 234 contigs with N50 = 574bp. Therefore, MEGAHIT results were used for further analysis. In total, GBM assembly contains 39 000 contigs longer than 500 nt and the largest contig is 14kb. The normal brain assembly contains 33 640 contigs longer than 500 nt, with largest contig reaching 37.5 kb.

### 3.3 Assembly annotation

The assembled contigs were annotated with the nucleotide collection database for Blast (nr/nt) at NCBI as well as Swiss‐Prot. Among the 95 642 contigs assembled from GBM samples, 93 228 can be annotated by nt database, 55 200 are annotated by nr database, and 47 070 contigs are annotated by Swiss‐Prot database. Only 959 contigs cannot be annotated by neither of the three databases (Figure 3A,C). Out of 71 771 contigs assembled from healthy brain samples, 69 255 can be annotated by nt database, 49 782 are annotated by nr database and 41 615 are annotated by Swiss‐Prot database, with only 369 contigs cannot be annotated (Figure 3B,D).

**FIGURE 4:**
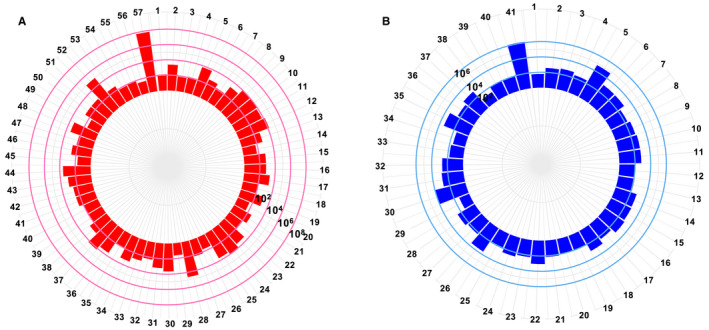
The reads abundance for the annotated contigs from Table 1. A, The GBM and B, normal brain. The *X*‐axis represents the name of contigs (Table 2), the *Y*‐axis represents the number of reads that can be mapped to the contigs, in log10 scale

**FIGURE 5:**
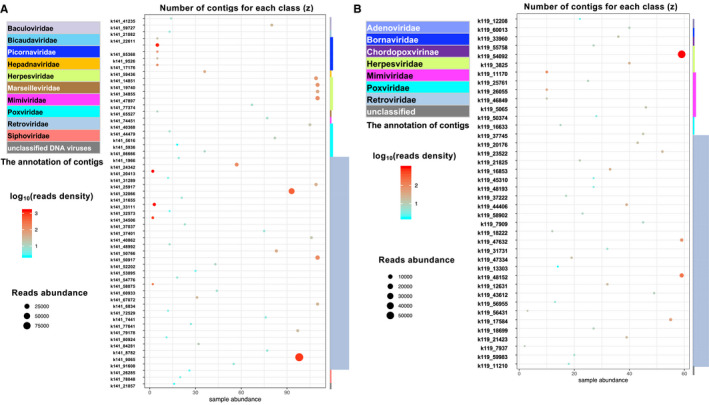
The distribution of virus contigs in different samples, (phage excluded). A, The GBM and B, normal brain. The *X*‐axis represents the number of samples that harbor these contigs. The *Y*‐axis list the individual contigs; the reads abundance is represented by the size of the dot; the color represents the reads density (reads number/sample numbers) in log 10 scale; the taxonomy of the annotated virus is presented on the right of the chart, with the *z*‐axis for the number of contigs for each order

**FIGURE 6:**
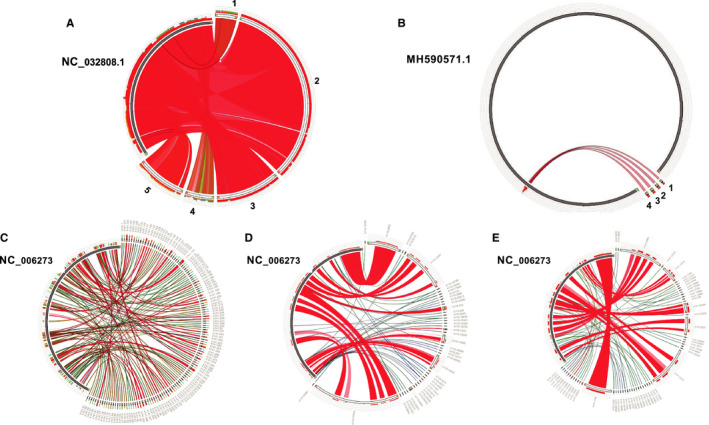
The assembled contigs from known viral infections and synteny analysis with their reference genomes. A, Wenzhou picorna‐like virus 2 strain. Contigs: 1: k141.22611 2: k141.83074; 3: k141.85368; 4: k141.9526; 5. k141.17176. B, Human gammaherpesvirus 4, reference genome accession: MH590571.1 contigs: 1: k141.19740 2: k141.34855 3: k141.14851 4: k141.47897. C, HCMV from seropositive healthy human samples D, HCMV from fetal lung fibroblast cells from naturally infection E, latent HCMV from hematopoietic cell

Of the annotated contigs over 500 bp long, 57 from GBM and 42 from healthy brain were identified as putative viral sequences of nonhuman origin (Table 1). Most of these contigs have a minimum read depth of 100 over the entire contig (Figure 4A,B, Table 2). Figure 5 shows the detailed information about these contigs. Most of these viral annotations can be characterized as retroviridae. Surprisingly, five contigs were annotated as a novel picornavirus previously identified from invertebrates.^84^ These viral contigs were detected in five GBM but none of the healthy brain samples. The synteny analysis shows that these five contigs can match up to more than 90% of the picorna‐like virus 2 reference genome (Figure 6A). This result suggests a possible cross species transmission of the virus.

**TABLE 1:** The annotated contigs with length > 500 bp by nr, nt, and Swiss‐Prot in GBM and normal brain. (A) The annotated virus from nr, nt, Swiss‐Prot in GBM. (B) The annotated virus from nr, nt, Swiss‐Prot in normal brain

Swiss‐Prot >333 AA	Swiss‐Prot 167‐333 AA	NR >333AA	NR 167‐333AA	NT >1000	NT 500‐1000
(A)					
k141_1966	k141_17176	k141_17176	k141_21057	k141_17176	k141_14851
k141_20413	k141_21082	k141_1966	k141_22611	k141_20413	k141_19740
k141_22611	k141_22611	k141_20413	k141_26285	k141_33111	k141_22611
k141_24342	k141_25917	k141_22611	k141_31289	k141_34506	k141_26285
k141_31655	k141_31289	k141_65527	k141_32573	k141_50766	k141_34855
k141_32066	k141_32573	k141_83074	k141_33111	k141_5616	k141_41235
k141_33111	k141_33111	k141_85368	k141_54776	k141_83074	k141_47897
k141_34506	k141_34506	k141_90342	k141_72529		k141_50766
k141_37037	k141_37401	k141_9526	k141_80924		k141_54776
k141_50766	k141_40368				k141_58075
k141_52202	k141_40862				k141_78048
k141_67072	k141_44479				k141_9526
k141_79178	k141_48992				
k141_83074	k141_50917				
k141_84281	k141_53095				
k141_85368	k141_54776				
k141_8782	k141_5936				
	k141_59436				
	k141_59727				
	k141_60933				
	k141_6834				
	k141_7441				
	k141_74451				
	k141_77374				
	k141_77641				
	k141_83074				
	k141_85368				
	k141_86666				
	k141_9065				
	k141_91608				
	k141_9526				
(B)					
k119_16633	k119_11170		k119_11210		k119_12208
k119_20176	k119_12162		k119_12208		k119_33960
k119_23522	k119_12208		k119_17584		k119_54092
k119_31731	k119_12631		k119_20176		
k119_47334	k119_13303		k119_23522		
k119_56431	k119_16853		k119_66335		
k119_58902	k119_17584		k119_7909		
k119_60013	k119_18222				
	k119_18699				
	k119_20176				
	k119_21423				
	k119_21825				
	k119_25761				
	k119_26055				
	k119_37222				
	k119_37745				
	k119_3825				
	k119_43612				
	k119_44406				
	k119_45310				
	k119_46849				
	k119_47632				
	k119_48152				
	k119_48193				
	k119_50374				
	k119_5065				
	k119_55758				
	k119_56955				
	k119_59983				
	k119_60013				
	k119_7909				
	k119_7937				

**TABLE 2:** The assembled contigs annotated as viral origin with number of mapped reads and labels presented in Figure 4

GBM assembly			Normal brain assembly		
Label	Contigs	Total reads	Label	Contigs	Total reads
1	k141_41235	128	1	k119_12208	60
2	k141_59727	2354	2	k119_60013	379
3	k141_21082	78	3	k119_33960	531
4	k141_22611	126	4	k119_55758	244
5	k141_83074	6389	5	k119_54092	58 879
6	k141_85368	442	6	k119_3825	1115
7	k141_9526	133	7	k119_11170	1076
8	k141_59436	1824	8	k119_25761	219
9	k141_14851	9209	9	k119_26055	443
10	k141_19740	11 153	10	k119_46849	151
11	k141_34855	6415	11	k119_5065	632
12	k141_47897	14 070	12	k119_50374	107
13	k141_77374	584	13	k119_16633	122
14	k141_65527	58	14	k119_37745	651
15	k141_74451	643	15	k119_20176	734
16	k141_17176	646	16	k119_23522	1084
17	k141_40368	2165	17	k119_21825	251
18	k141_44479	104	18	k119_16853	1018
19	k141_5616	1184	19	k119_45310	90
20	k141_5936	42	20	k119_48193	108
21	k141_86666	377	21	k119_37222	138
22	k141_1966	113	22	k119_44406	1131
23	k141_20413	3011	23	k119_58902	174
24	k141_24342	8541	24	k119_7909	329
25	k141_25917	4573	25	k119_18222	124
26	k141_31289	50	26	k119_47632	4153
27	k141_31655	151	27	k119_31731	172
28	k141_32066	41 520	28	k119_47334	342
29	k141_32573	39	29	k119_13303	23
30	k141_33111	4324	30	k119_48152	9792
31	k141_34506	1697	31	k119_12631	608
32	k141_37037	183	32	k119_43612	461
33	k141_37401	512	33	k119_56955	61
34	k141_40862	2007	34	k119_56431	38
35	k141_48992	110	35	k119_17584	2603
36	k141_50766	3945	36	k119_18699	246
37	k141_50917	15 136	37	k119_21423	734
38	k141_52202	472	38	k119_7937	21
39	k141_53095	108	39	k119_59983	114
40	k141_54776	116	40	k119_11210	82
41	k141_58075	338	41	k119_66335	1 280 118
42	k141_60933	342			
43	k141_67072	1032			
44	k141_6834	3787			
45	k141_72529	54			
46	k141_7441	418			
47	k141_77641	147			
48	k141_79178	2654			
49	k141_80924	43			
50	k141_84281	481			
51	k141_8782	475			
52	k141_9065	95 447			
53	k141_91608	348			
54	k141_26285	77			
55	k141_78048	83			
56	k141_21057	40			
57	k141_90342	64 234 471			

We also identified four contigs (k141_19740 (length = 664); k141_34855 (length = 739); k141_14851 (length = 753); k141_47897 (length = 501)) that were annotated as EBV, the only herpes virus to be found with moderate length of contigs. However, the synteny analysis showed that they are mapped to the same small region of the EBV reference genome (Figure 6B). In contrast, the synteny analysis of the presence of herpes virus in positive control showed significant number of contigs homologous to the HCMV reference genome (Figure 1). Significant homology over large genomic area is also observed in HCMV contigs from CMV seropositive healthy human samples (Figure 6C), fetal lung fibroblast cells from naturally infected people (Figure 6D), and HCMV latent hematopoietic cell (Figure 6E). In addition, READSCAN analysis of GBM virome does not support the presence of herpesviruses in GBM despite of few reads in few samples appeared to be mapped to a small proportion of the viral genome (Supplemental 3).^64^ Therefore, both the contig assembly and sequence reads mapping from our analysis do not support the presence of EVB and other herpesviruses in GBM. However, our analysis cannot rule out the presence of latent herps virus whose genomic DNA is inserted into the genome of GBM tumor cells.

### 3.4 Novel contig antigen prediction

**FIGURE 7:**
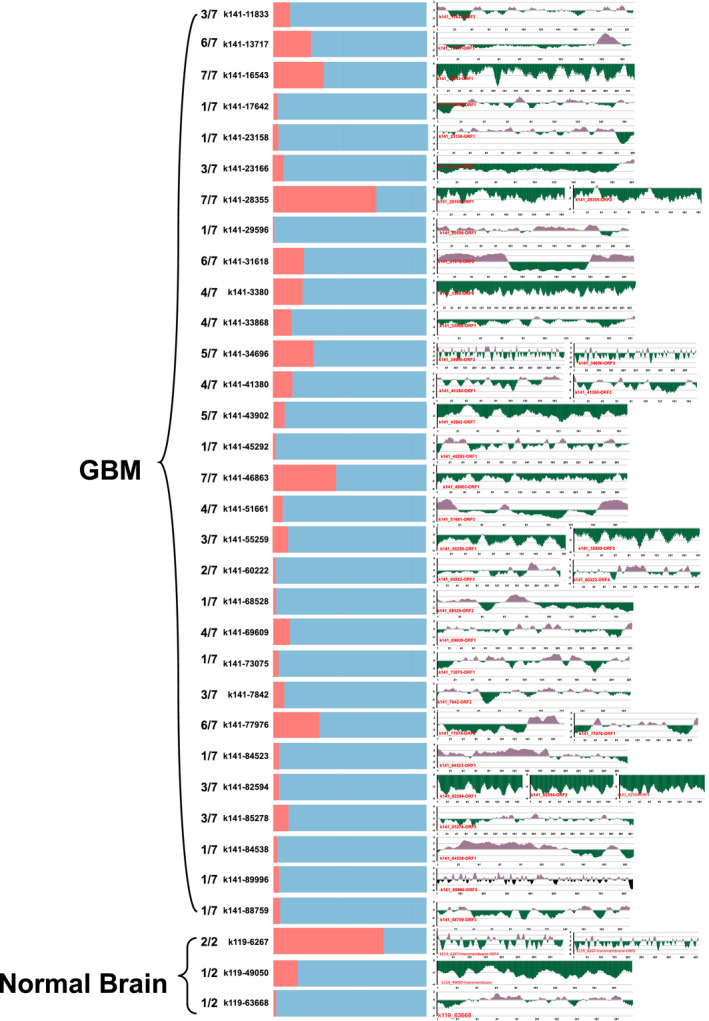
The antibody epitope prediction and sample distribution. The antibody epitope prediction results are on the right, *Y*‐axis represents the score of the antigen prediction and the *X*‐axis represents the position of the predicted open reading frame. On the left are the proportion of samples (blue) that harbor this contig out of 110 GBM and 57 normal brain tissues. The number represents the proportion of projects that harbor the contigs

For unannotated 959 contigs from GBM and 369 from healthy brain (Figure 3C,D), we performed phylogenetic analysis to group them into three major clusters (Supplemental 4A, 4B). ORF was predicted for each contig longer than 500bp. The resulting protein sequences from these predicted ORF were subject to TMHMM v2.0 (http://www.cbs.dtu.dk/services/TMHMM/) analysis to predict the transmembrane domains. Significant transmembrane domains were found in 31 unknown contigs from GBM and three unknown contigs from health brain. Among these transmembrane contigs, we found that the linear B‐cell epitopes were enriched and analyzed. Some of the contigs, such as k141_31618 assembled from 22 out of 110 GBM samples and k141_77976 from 33 of GBM samples, contains putative antigen epitopes (Figure 7). If real and validated by experiments, these contigs can potentially be recognized by immune system and used as targets for drug development.

## 4 DISCUSSION

As the most lethal type of cancer, GBM kills thousands every year. Although many studies have investigated the risk factors of GBM, our knowledge of their etiology is still lacking.^9^, ^11^, ^12^, ^13^, ^14^, ^85^ Emerging evidence suggests that viral infection can cause tumors. For GBM, the main focus was on HCMV, with a small number of studies on other viruses such as EBV^86^ or HPV^87^ by amplifying viral genome segments. However, the presence and association of virus with GBM is not firmly established and an un‐biased data‐driven approach to investigate the virome in human brain is needed. Analyzing virome in GBM can provide insight on etiology of GBM, and maybe it's unexplainable relationship with other neurological disorder such as Alzheimer's disease.

Next‐generation sequencing technologies had been successfully applied to characterize the virome in various human tissues such as skin and blood.^88^, ^89^, ^90^ Traditional methods for viral detection are based on aligning short sequence reads to the reference viral genome sequences with commonly used software such as PathSequation^91^ or RINs.^92^ However, these methods could suffer from false positive results where short sequence reads can be congregated in highly repetitive regions. Besides, some reference viral genomes may also contain artificial sequences.^54^ Our approach avoided this drawback by first mapping sequence reads to the human genome to filter out human protein‐coding genes and other highly repetitive elements such as human endogenous retrovirus or transposable element sequences. The unmapped reads containing viral sequences were then assembled into relative longer contigs. Our study is the first to explore the GBM virome in an assembly annotation approach, and indeed we identified contigs that match viral sequences. Among them, most were retrovirus sequences, probably due to the close relationship of the retrovirus with human transposable elements.^93^ We also found extensive presence of phage sequences in both GBM and healthy brain. Even though it is possible that they come from the gut,^94^ previous studies often consider them from bacterial infections contaminated by the commercial phiX174.^88^, ^95^, ^96^

It is surprising to find the sequences of a Picornavirus in five GBM samples (Supplemental 5), as this virus was first reported in invertebrate.^9^, ^10^, ^11^, ^85^ However, it is unlikely due to sample contamination or sequence mismatches as the five assembled contigs cover more than 90% of the reference genome of the virus. Picornaviruses are small, single‐stranded positive RNA viruses infecting a wide range of hosts. Given that some viruses infect their hosts ranging from plants to animals,^97^ the ubiquitous presence of the Picornaviruses suggests a complex nature of virosphere and an extensive horizontal genetic exchanges of viral genomics.^98^ Our finding also indicates that this virus could be a new candidate for oncolytic viral therapy since several other picornaviruses had been proven to have the oncolytic potentials. For example, a recombinant oncolytic poliovirus, PVSRIPO has demonstrated to be oncolytic in a wide range of brain cancer cell lines such as GBM cell lines^99^ or astrocytomas cancer cell lines.^100^, ^101^ Other attenuated polioviruses such as incompetent poliovirus 1 (PV1) replicons have also shown cytotoxicity against various tumors and promising results in prolong survival of GBM mouse models.^102^ Taken together, the detection of picornavirus in the GBM but not healthy samples suggests the potential of the discovered picornavirus as a candidate to engineer future oncolytic virus.^103^

The presence of EBV in gliomas has always been controversy.^86^ Consistent with some of the previous studies,^34^, ^35^, ^39^, ^52^, ^104^ our results suggest that EBV is absent from gliomas. In addition, contig segments matching herpes virus sequences may come from homologous sequences. However, one possibility we cannot rule out is that the herpes virus is in latent in GBM or inserted into the human genome in various tissues that cannot be captured by RNA‐seq.

A number of contigs cannot be annotated by any databases we used. It is possible that those contigs are artificial or formed from artificial sequences such as vectors or contaminations. However, we observed that various samples from different projects have reads that can cover more than 60% of the contig. For example, over 60% of the length of contig k141‐31618 can be covered by the reads originated from 22 studies from six out of seven projects in the GBM group, making it evident that contigs like this are not contaminations but rather originated from a valid source. Transmembrane analysis and antibody epitope prediction show that significant amount of those contig sequences has antibody epitope sequence signature, suggesting a potential to be used as drug targets for cancer immune therapy.

## CONFLICT OF INTEREST

The authors declare that they have no competing interests.

## AUTHOR CONTRIBUTIONS

ZY, WJZ, and ZA conceived and designed the study, ZY and LZ performed the analysis, XY, LZ, NZ, ZA and WJZ made revisions, ZA and WJZ supervised the project. All authors support the publication of the manuscript.

## Supporting information

Supplementary Material

Supplementary Material

Supplementary Material

Supplementary Material

Supplementary Material

## Data Availability

We searched the NCBI Sequence Read Archive (SRA) (https://www.ncbi.nlm.nih.gov/sra), Gene Expression Omnibus (GEO) (https://www.ncbi.nlm.nih.gov/geo/), and literatures to collect the next‐generation sequencing (NGS) studies relating to GBM and normal brain tissues, as well as samples infected with known virus as “positive controls” to test our assembly approaches. The raw RNA‐seq fastq files from Illumina platform were downloaded from SRA database, and the list of accessions for the source data is shown in the Supplemental File 1.
