# Supplementary material for: Virome assembly and annotation in brain tissue based on next‐generation sequencing
Source: Cancer Med. 2020 Aug 1;9(18):6776–90. doi: 10.1002/cam4.3325 (PMC7520322; doi:10.1002/cam4.3325)
Supplement: Supplementary file 1 — Supplementary Material [file CAM4-9-6776-s001.docx]

**Supplemental 1. The accessions for sample from GBM, normal brain** Accession numbers for the SRA runs and experiments are provided

| **Samples** | **Run Accession** | **Experiment Accession** | **BioProject** |
| --- | --- | --- | --- |
| **GBM** | ERR1018443 | ERX1097381 | PRJEB10881 |
|  | ERR1018444 | ERX1097382 |  |
|  | ERR1018445 | ERX1097383 |  |
|  | ERR1018446 | ERX1097384 |  |
|  | ERR1018447 | ERX1097385 |  |
|  | ERR1018448 | ERX1097386 |  |
|  | ERR1018449 | ERX1097387 |  |
|  | ERR1018450 | ERX1097388 |  |
|  | ERR1018451 | ERX1097389 |  |
|  | ERR1018452 | ERX1097390 |  |
|  | ERR1018453 | ERX1097391 |  |
|  | ERR1018454 | ERX1097392 |  |
|  | ERR1018455 | ERX1097393 |  |
|  | ERR1018456 | ERX1097394 |  |
|  | ERR1018457 | ERX1097395 |  |
|  | ERR1018458 | ERX1097396 |  |
|  | ERR1018459 | ERX1097397 |  |
|  | ERR1018460 | ERX1097398 |  |
|  | SRR1521352 | SRX657979 | PRJNA255754  (1) |
|  | SRR1521353 | SRX657980 |  |
|  | SRR1521354 | SRX657981 |  |
|  | SRR1521355 | SRX657982 |  |
|  | SRR1521356 | SRX657983 |  |
|  | SRR1521357 | SRX657984 |  |
|  | SRR1521358 | SRX657985 |  |
|  | SRR1521359 | SRX657986 |  |
|  | SRR1521360 | SRX657987 |  |
|  | SRR1521361 | SRX657988 |  |
|  | SRR1521362 | SRX657989 |  |
|  | SRR1521363 | SRX657990 |  |
|  | SRR1521364 | SRX657991 |  |
|  | SRR1521365 | SRX657992 |  |
|  | SRR1521366 | SRX657993 |  |
|  | SRR1521367 | SRX657994 |  |
|  | SRR1521368 | SRX657995 |  |
|  | SRR1521369 | SRX657996 |  |
|  | SRR1521370 | SRX657997 |  |
|  | SRR1521371 | SRX657998 |  |
|  | SRR1521372 | SRX657999 |  |
|  | SRR1521373 | SRX658000 |  |
|  | SRR1521374 | SRX658001 |  |
|  | SRR1521375 | SRX658002 |  |
|  | SRR1521376 | SRX658003 |  |
|  | SRR1521377 | SRX658004 |  |
|  | SRR1521378 | SRX658005 |  |
|  | SRR1521379 | SRX658006 |  |
|  | SRR1521380 | SRX658007 |  |
|  | SRR1521381 | SRX658008 |  |
|  | SRR1521382 | SRX658009 |  |
|  | SRR1521383 | SRX658010 |  |
|  | SRR1521384 | SRX658011 |  |
|  | SRR1521385 | SRX658012 |  |
|  | SRR1521386 | SRX658013 |  |
|  | SRR1521387 | SRX658014 |  |
|  | SRR1521388 | SRX658015 |  |
|  | SRR1521389 | SRX658016 |  |
|  | SRR1521390 | SRX658017 |  |
|  | SRR1521391 | SRX658018 |  |
|  | SRR1521392 | SRX658019 |  |
|  | SRR1521393 | SRX658020 |  |
|  | SRR1521394 | SRX658021 |  |
|  | SRR1521395 | SRX658022 |  |
|  | SRR1521396 | SRX658023 |  |
|  | SRR1521397 | SRX658024 |  |
|  | SRR1521398 | SRX658025 |  |
|  | SRR1521399 | SRX658026 |  |
|  | SRR1521400 | SRX658027 |  |
|  | SRR1521401 | SRX658028 |  |
|  | SRR1521402 | SRX658029 |  |
|  | SRR1521403 | SRX658030 |  |
|  | SRR1521404 | SRX658031 |  |
|  | SRR1521405 | SRX658032 |  |
|  | SRR1521406 | SRX658033 |  |
|  | SRR1521407 | SRX658034 |  |
|  | SRR1521408 | SRX658035 |  |
|  | SRR1521409 | SRX658036 |  |
|  | SRR1521410 | SRX658037 |  |
|  | SRR1521411 | SRX658038 |  |
|  | SRR1521412 | SRX658039 |  |
|  | SRR1521413 | SRX658040 |  |
|  | SRR1521414 | SRX658041 |  |
|  | SRR1521415 | SRX658042 |  |
|  | SRR1521416 | SRX658043 |  |
|  | SRR1521417 | SRX658044 |  |
|  | SRR1521418 | SRX658045 |  |
|  | SRR1521419 | SRX658046 |  |
|  | SRR1521420 | SRX658047 |  |
|  | SRR1521421 | SRX658048 |  |
|  | SRR1521422 | SRX658049 |  |
|  | SRR1521423 | SRX658050 |  |
|  | SRR1521424 | SRX658051 |  |
|  | SRR1521425 | SRX658052 |  |
|  | SRR1521426 | SRX658053 |  |
|  | SRR1633194 | [SRX745136](https://www.ncbi.nlm.nih.gov/sra/SRX745136) | PRJNA265085  (2) |
|  | SRR1633195 | [SRX745137](https://www.ncbi.nlm.nih.gov/sra/SRX745137) |  |
|  | SRR1825693 | SRX897035 | PRJNA276922  (3) |
|  | SRR1825697 | SRX897040 |  |
|  | SRR2226702 | SRX1177329 | PRJNA294444  (4) |
|  | SRR2226703 | SRX1177330 |  |
|  | SRR2226704 | SRX1177331 |  |
|  | SRR2226705 | SRX1177332 |  |
|  | SRR2226706 | SRX1177333 |  |
|  | SRR2226707 | SRX1177334 |  |
|  | SRR2226738 | SRX1177365 |  |
|  | SRR4241104 | SRX2162078 | PRJNA342811 |
|  | SRR4241106 | SRX2162080 |  |
|  | SRR4241108 | SRX2162082 |  |
|  | SRR4241110 | SRX2162084 |  |
|  | SRR5635314 | SRX2875070 | PRJNA388704 |
|  | SRR5635315 | SRX2875071 |  |
|  | SRR5635317 | SRX2875072 |  |

| **Samples** | **Run Accession** | **Experiment Accession** | **BioProject** |
| --- | --- | --- | --- |
| **Normal Brain** | ERR1474899 | ERX1546036 | PRJEB14617 |
|  | ERR1474900 | ERX1546037 |  |
|  | ERR1474901 | ERX1546038 |  |
|  | ERR1474902 | ERX1546039 |  |
|  | ERR1474903 | ERX1546040 |  |
|  | ERR1474904 | ERX1546041 |  |
|  | ERR1474905 | ERX1546042 |  |
|  | ERR1474906 | ERX1546043 |  |
|  | ERR1474907 | ERX1546044 |  |
|  | ERR1474908 | ERX1546045 |  |
|  | SRR1521427 | SRX658054 | PRJNA255754  (1) |
|  | SRR1521428 | SRX658055 |  |
|  | SRR1521429 | SRX658056 |  |
|  | SRR1521430 | SRX658057 |  |
|  | SRR1521431 | SRX658058 |  |
|  | SRR1521432 | SRX658059 |  |
|  | SRR1521433 | SRX658060 |  |
|  | SRR1521434 | SRX658060 |  |
|  | SRR1521435 | SRX658060 |  |
|  | SRR1521436 | SRX658061 |  |
|  | SRR1521437 | SRX658062 |  |
|  | SRR1521438 | SRX658063 |  |
|  | SRR1521439 | SRX658064 |  |
|  | SRR1521440 | SRX658065 |  |
|  | SRR1521441 | SRX658066 |  |
|  | SRR1521442 | SRX658067 |  |
|  | SRR1521443 | SRX658068 |  |
|  | SRR1521444 | SRX658069 |  |
|  | SRR1521445 | SRX658070 |  |
|  | SRR6145415 | SRX3257506 |  |
|  | SRR6145416 | SRX3257507 |  |
|  | SRR6145417 | SRX3257508 |  |
|  | SRR6145418 | SRX3257509 |  |
|  | SRR6145419 | SRX3257510 |  |
|  | SRR6145420 | SRX3257511 |  |
|  | SRR6145421 | SRX3257512 |  |
|  | SRR6145422 | SRX3257513 |  |
|  | SRR6145423 | SRX3257514 |  |
|  | SRR6145424 | SRX3257515 |  |
|  | SRR6145425 | SRX3257516 |  |
|  | SRR6145426 | SRX3257517 |  |
|  | SRR6145427 | SRX3257518 |  |
|  | SRR6145428 | SRX3257519 |  |
|  | SRR6145429 | SRX3257520 |  |
|  | SRR6145430 | SRX3257521 |  |
|  | SRR6145431 | SRX3257522 |  |
|  | SRR6145432 | SRX3257523 |  |
|  | SRR6145433 | SRX3257524 |  |
|  | SRR6145434 | SRX3257525 |  |
|  | SRR6145435 | SRX3257526 |  |
|  | SRR6145436 | SRX3257527 |  |
|  | SRR6145437 | SRX3257528 |  |
|  | SRR6145438 | SRX3257529 |  |
|  | SRR6145439 | SRX3257530 |  |
|  | SRR6145440 | SRX3257531 |  |
|  | SRR6145441 | SRX3257532 |  |
|  | SRR6145442 | SRX3257533 |  |
|  | SRR6145443 | SRX3257534 |  |
|  | SRR6145444 | SRX3257535 |  |

**Reference**

1. Gill BJ, Pisapia DJ, Malone HR, Goldstein H, Lei L, Sonabend A, et al. MRI-localized biopsies reveal subtype-specific differences in molecular and cellular composition at the margins of glioblastoma. Proceedings of the National Academy of Sciences. 2014;111(34):12550-5.

2. Stathias V, Pastori C, Griffin TZ, Komotar R, Clarke J, Zhang M, et al. Identifying glioblastoma gene networks based on hypergeometric test analysis. PloS one. 2014;9(12):e115842.

3. Park C-K, Park I, Lee S, Sun C-H, Koh Y, Park S-H, et al. Genomic dynamics associated with malignant transformation in IDH1 mutated gliomas. Oncotarget. 2015;6(41):43653.

4. Liu F, Hon GC, Villa GR, Turner KM, Ikegami S, Yang H, et al. EGFR mutation promotes glioblastoma through epigenome and transcription factor network remodeling. Molecular cell. 2015;60(2):307-18.
