# Supplementary material for: Virome assembly and annotation in brain tissue based on next‐generation sequencing
Source: Cancer Med. 2020 Aug 1;9(18):6776–90. doi: 10.1002/cam4.3325 (PMC7520322; doi:10.1002/cam4.3325)
Supplement: Supplementary file 2 — Supplementary Material [file CAM4-9-6776-s002.docx]

**Supplemental 2. The accessions for sample with known viral infections.** Human cytomegalovirus; Enterobacteria phage phiX174; Hepatitis B virus; Zika virus; Tick-borne Encephalitis virus; Influenza A virus H1N1; Human cytomegalovirus in natural infection and experimental latency. The project accessions and their references are provided on the header of the table.

| **PRJEB30943 (1)** | **Run Accession** | **Experiment Accession** |
| --- | --- | --- |
| **Human cytomegalovirus** | ERR3089098 | ERX3149926 |

| **PRJNA433861** | **Run Accession** | **Experiment Accession** |
| --- | --- | --- |
| **Enterobacteria phage phiX174** | SRR6755978 | SRX3728607 |

| **PRJNA338726** | **Run Accession** | **Experiment Accession** |  |
| --- | --- | --- | --- |
| **Hepatitis B virus** | SRR4021807 | SRX2014052 | |
|  | SRR4021808 | SRX2014054 | |
|  | SRR4021809 | SRX2014055 | |

| **PRJEB14936** | **Run Accession** | **Experiment Accession** |
| --- | --- | --- |
| **Zika virus** | ERR1549324 | ERX1620094 |
|  | ERR1549325 | ERX1620095 |
|  | ERR1549326 | ERX1620096 |
|  | ERR1549327 | ERX1620097 |
|  | ERR1549329 | ERX1620099 |

| **PRJEB14767** | **Run Accession** | **Experiment Accession** |
| --- | --- | --- |
| **Tick-borne Encephalitis virus** | ERR1527186 | ERX1598083 |

| **PRJNA534263**  (2) | **Run Accession** | **Experiment Accession** |
| --- | --- | --- |
| **Influenza A virus H1N1** | SRR8945480 | SRX5725612 |

| **PRJNA389726**  (3) | **Run** | **Experiment Accession** |
| --- | --- | --- |
| **Fetal lung fibroblast cells from naturally infection** | SRR5660008 | SRX2896338 |
|  | SRR5660009 | SRX2896339 |
|  | SRR5660010 | SRX2896340 |
|  | SRR5660011 | SRX2896341 |
|  | SRR5660012 | SRX2896342 |
|  | SRR5660013 | SRX2896343 |
|  | SRR5660014 | SRX2896344 |
|  | SRR5660015 | SRX2896345 |
|  | SRR5660016 | SRX2896346 |
|  | SRR5660017 | SRX2896347 |
|  | SRR5660018 | SRX2896348 |
|  | SRR5660019 | SRX2896349 |
|  | SRR5660020 | SRX2896350 |
|  | SRR5660021 | SRX2896351 |
|  | SRR5660022 | SRX2896352 |
|  | SRR5660023 | SRX2896353 |
| **HCMV latent hematopoietic cell** | SRR5660024 | SRX2896354 |
|  | SRR5660025 | SRX2896355 |
|  | SRR5660026 | SRX2896356 |
|  | SRR5660027 | SRX2896357 |
|  | SRR5660028 | SRX2896358 |
|  | SRR5660029 | SRX2896359 |
|  | SRR5660030 | SRX2896360 |
|  | SRR5660031 | SRX2896361 |
|  | SRR5660032 | SRX2896362 |
|  | SRR5660033 | SRX2896363 |
|  | SRR5660034 | SRX2896364 |
|  | SRR5660035 | SRX2896365 |
|  | SRR5660036 | SRX2896366 |
|  | SRR5660037 | SRX2896367 |
|  | SRR5660038 | SRX2896368 |
|  | SRR5660039 | SRX2896369 |
|  | SRR5660040 | SRX2896370 |
|  | SRR5660041 | SRX2896371 |
|  | SRR5660042 | SRX2896372 |
|  | SRR5660043 | SRX2896373 |
|  | SRR5660044 | SRX2896374 |
|  | SRR5660045 | SRX2896375 |
|  | SRR5660046 | SRX2896376 |
|  | SRR5660047 | SRX2896377 |
|  | SRR5660048 | SRX2896378 |
|  | SRR5660049 | SRX2896379 |
|  | SRR6133833 | SRX3255200 |
|  | SRR6133834 | SRX3255201 |
|  | SRR6133835 | SRX3255202 |
|  | SRR6133836 | SRX3255203 |
| **Healthy human samples with CMV seropositive** | SRR5660050 | SRX2896380 |
|  | SRR5660051 | SRX2896381 |
|  | SRR5660052 | SRX2896382 |

**Reference**

1. Krenzlin H, Behera P, Lorenz V, Passaro C, Zdioruk M, Nowicki MO, et al. Cytomegalovirus promotes murine glioblastoma growth via pericyte recruitment and angiogenesis. The Journal of clinical investigation. 2019;129(4).

2. Chambers BS, Heaton BE, Rausch K, Dumm RE, Hamilton JR, Cherry S, et al. DNA mismatch repair is required for the host innate response and controls cellular fate after influenza virus infection. Nature microbiology. 2019;4(11):1964-77.

3. Cheng S, Caviness K, Buehler J, Smithey M, Nikolich-Žugich J, Goodrum F. Transcriptome-wide characterization of human cytomegalovirus in natural infection and experimental latency. Proceedings of the National Academy of Sciences. 2017;114(49):E10586-E95.
