# Supplementary figures and images for: Virome assembly and annotation in brain tissue based on next‐generation sequencing
Source: Cancer Med. 2020 Aug 1;9(18):6776–90. doi: 10.1002/cam4.3325 (PMC7520322; doi:10.1002/cam4.3325)

A

Tree scale: 0.1

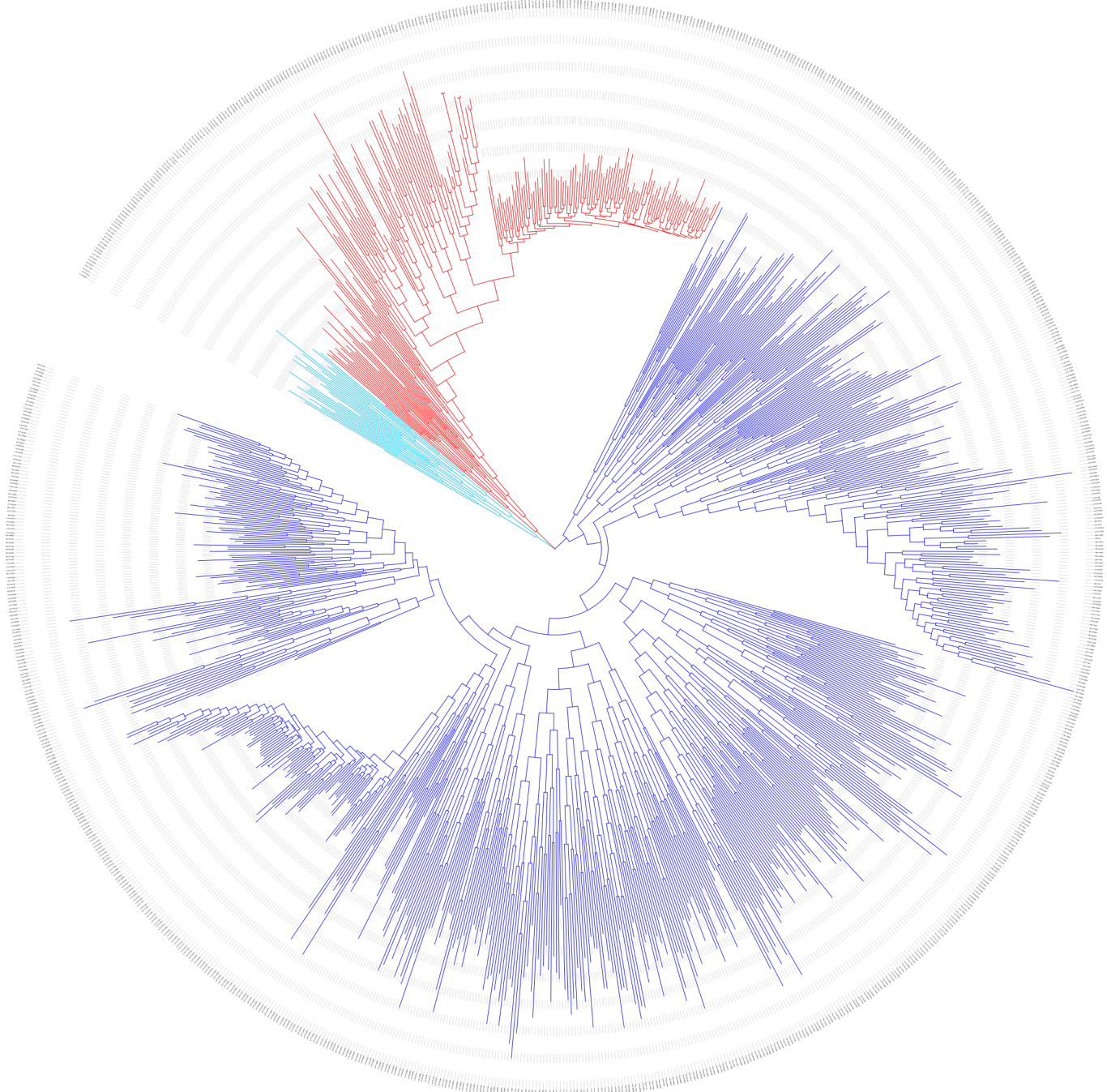

B

Tree scale: 0.1

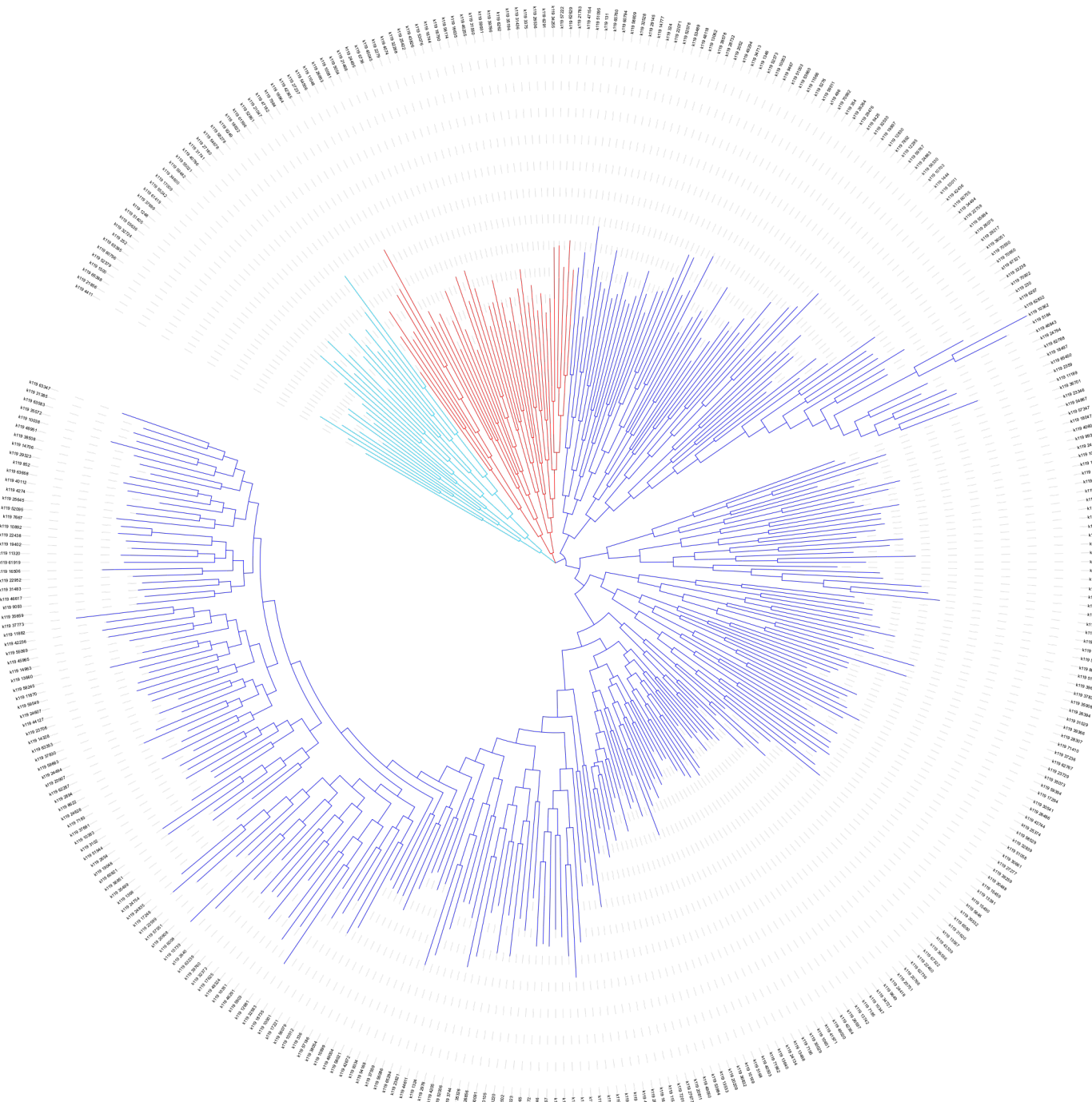

Supplement: Supplementary file 4 — Supplementary Material [file CAM4-9-6776-s004.pdf]

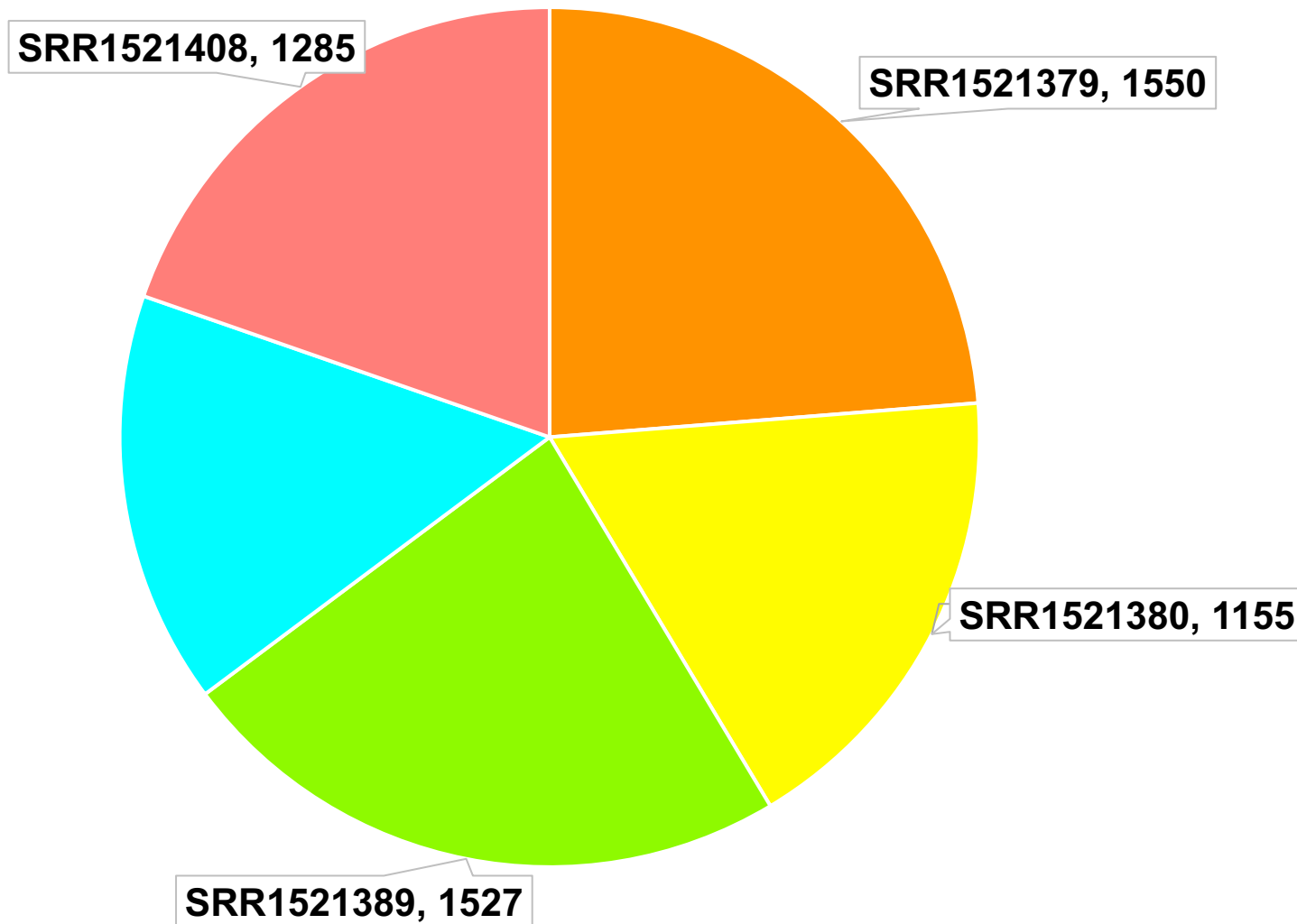

■ **SRR1521379**

■ **SRR1521380**

■ **SRR1521389**

■ **SRR1521407**

■ **SRR1521408**

Supplement: Supplementary file 5 — Supplementary Material [file CAM4-9-6776-s005.pdf]
